# Dr. Parrish on Dysentery

**Published:** 1851-10

**Authors:** 


### ECLECTIC DEPARTMENT,

### AND SPIRIT OF THE MEDICAL PERIODICAL PRESS.

---

*Dr. Parrish on Dysentery.*—We copy the following extract from an interesting article by Dr. Parrish, editor of the New Jersey Med. Reporter. —EDITOR.

In those cases which presented none of the complications referred to, opium was employed as a principal remedy; and we are well satisfied that this drug possesses the power to control and cure dysentery in most instances. Given largely, it will cut short the disease in its incipient stage, in a very short time. Our own practice has been, to administer to an adult, two, and even three, grains in cases of extreme suffering, at a single dose; to insist upon perfect rest in the recumbent posture, to elevate the hips by pillows, with a view of diverting the current of the circulation from the rectum, to allow but little nourishment except that of the blandest kind, as gum water, linseed gruel, &c., and we have generally been satisfied with its effects. Of course, if there is reason to believe that hardened fæces, or crude ingesta, are lodged in the bowels, they are removed by appropriate cathartics; but we believe the great and prominent indication in this disease, is to keep the bowels at rest after they are emptied; and then, by position, to prevent the excessive flow of blood to the seat of pain, while opium is administered to relieve spasm and tenesmus, control the circulation, strengthen the nervous system, and produce sleep, all of which indications it is capable of fulfilling.

We have sometimes combined other remedies with opium; but the fact that no other medicine, when employed alone, will relieve the distressing symptoms—that to make it effectual it must be united with opium in some of its forms—is sufficient to induce the inquiry whether after all opium may not be relied on, itself. But are not the secretions vitiated? Must we not use an alterative? Let us decide, then, which of the secretions is deranged, and what is the cause and nature of its change; for if we are to use an alterative, we must know what requires to be altered. Experience has taught us that the bladder, urethra, stomach, and liver, sometimes sympathize so freely with the diseased intestine as to give rise to various complications depending upon the seat of functional derangement; so much so, that the biliary, gastric, and renal secretions become modified in their appearance and properties, and seem to call for special treatment; but if they are the results of the pathological condition which marks the disease, and dependent merely for their existence upon the sympathetic relations which are sustained between the different organs of the body, is it not more in accordance with sound principles of science to relieve the secondary symptoms, by removing the primary cause from which they originate? If, then, the function of the liver, for example, is so disturbed by the intestinal disease as to create either a diminution or increase in the quantity of bile secreted, or if this fluid should from the same cause, become changed in its character, why give calomel or any other medicine, simply with a view of altering the properties of the particular secretion, when the same result would flow, as a matter of course, from the removal of irritation, and relief of pain, heat, and tenesmus in the bowel? By the same rule we should give diuretics to stimulate the kidneys and bladder, when from sympathy, they fail to perform their functions. We do not urge any objection to the rule, except the fact that these combinations are apt to increase the irritation of the colon and rectum, even when guarded by opium, if continued for any considerable length of time. When the skin is hot and dry, we have found in our experience, the most grateful remedy to the patient, and we believe, one equally successful with most internal remedies, to be sponging the surface of the body with cold water and alcohol, whisky, or some convenient alcoholic liquor, to be followed by friction with a dry, coarse towel. In the adynamic form of the disease, or in the latter stage of ordinary cases where stimulation is demanded, we have seen the most signal benefit follow the administration of the following mixture:

℞. Aqua Camph. f℥iv.
Pv. G. Acac. ʒij.
Chloroform gtt. lx.
Tinct. Opii. fʒiss.
M. Signa. A tablespoonful every hour till relieved.

Under the use of this remedy we have seen in protracted cases of the disease, warmth return to the surface, and vigor to the pulse, in a few hours, when there seemed but little hope of permanent reaction. We cannot, therefore, fail to believe that the opiate and stimulating plan of treatment is that which is most likely to be successful in the treatment of the epidemic form of dysentery, particularly in malarious districts.

